# Thrombotic and Atherogenetic Predisposition in Polyglobulic Donors

**DOI:** 10.3390/biomedicines10040888

**Published:** 2022-04-12

**Authors:** Nikola Slaninova, Iveta Bryjova, Zenon Lasota, Radmila Richterova, Jan Kubicek, Martin Augustynek, Ayan Seal, Ondrej Krejcar, Antonino Proto

**Affiliations:** 1 Department of Cybernetics and Biomedical Engineering, VŠB—Technical University of Ostrava, 17. listopadu 2172/15, 708 00 Ostrava–Poruba, Czech Republic; nikola.slaninova.st@vsb.cz (N.S.); bryjatronic@gmail.com (I.B.); martin.augustynek@vsb.cz (M.A.); antonino.proto@unife.it (A.P.); 2 Blood Donor Center, tr. T. G. Masaryka 495, 738 01 Frydek-Mistek, Czech Republic; z.lasota@krevnicentrum.cz (Z.L.); r.richterova@krevnicentrum.cz (R.R.); 3 Department of Computer Science & Engineering, PDPM Indian Institute of Information Technology, Design and Manufacturing, Jabalpur 482005, India; ayan@iiitdmj.ac.in; 4 Center for Basic and Applied Research, Faculty of Informatics and Management, University of Hradec Kralove, Hradecka 1249, 500 03 Hradec Kralove, Czech Republic; ondrej.krejcar@uhk.cz

**Keywords:** *JAK2*, mutation, polycythemia vera, secondary polyglobulia

## Abstract

This work analyses the results of research regarding the predisposition of genetic hematological risks associated with secondary polyglobulia. The subjects of the study were selected based on shared laboratory markers and basic clinical symptoms. *JAK2* (Janus Kinase 2) mutation negativity represented the common genetic marker of the subjects in the sample of interest. A negative *JAK2* mutation hypothetically excluded the presence of an autonomous myeloproliferative disease at the time of detection. The parameters studied in this work focused mainly on thrombotic, immunological, metabolic, and cardiovascular risks. The final goal of the work was to discover the most significant key markers for the diagnosis of high-risk patients and to exclude the less important or only complementary markers, which often represent a superfluous economic burden for healthcare institutions. These research results are applicable as a clinical guideline for the effective diagnosis of selected parameters that demonstrated high sensitivity and specificity. According to the results obtained in the present research, groups with a high incidence of mutations were evaluated as being at higher risk for polycythemia vera disease. It was not possible to clearly determine which of the patients examined had a higher risk of developing the disease as different combinations of mutations could manifest different symptoms of the disease. In general, the entire study group was at risk for manifestations of polycythemia vera disease without a clear diagnosis. The group with less than 20% incidence appeared to be clinically insignificant for polycythemia vera testing and thus there is a potential for saving money in mutation testing. On the other hand, the *JAK V617F* (somatic mutation of *JAK2*) parameter from this group should be investigated as it is a clear exclusion or confirmation of polycythemia vera as the primary disease.

## 1. Introduction

Erythrocytes are one of the most important cells in the body due to their ability to transfer blood gases. They are nucleusless, biconcave-shaped blood elements formed in the red bone marrow, carrying hemoglobin. The life span is around 120 days. The average number of red blood cells in men is 4.3–5.7 1012/L in women it is 3.8–4.9 1012/L. Some men have a sustained increase in hemoglobin levels without a diagnosis of the chronic progressive clonal myeloproliferative disease polycythemia vera (*PV*). This disease is characterized by a *JAK V617F* mutation and a chronic increase in erythrocyte production as a primary disease. This paper focuses on the evaluation of hematological blood tests in patients prone to venous thrombosis and atherosclerosis with higher erythrocyte mass values.

Deep vein thrombosis is very difficult to diagnose and sometimes presents without significant difficulties. Most often it manifests itself as an embolism, or blockage of the artery. It is a partial or complete blockage of a deep vein by a blood clot, most often in the lower limb, very often leading to a second form of thromboembolic disease. It is most often recognized by unilateral swelling of the calf or the whole leg.

Thromboembolism is a condition where a blood clot has broken loose into the bloodstream and is closing a blood vessel. It can occur in venous system (venous thromboembolism) or in arteries (arterial thromboembolism).

**Figure 1:**
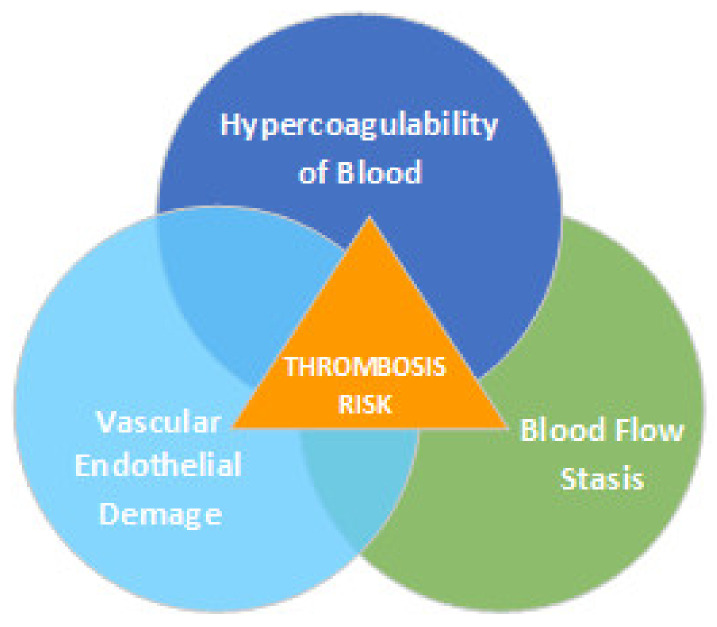
Virchow’s triad, which describes the three categories of factors in thrombosis.

Deep vein thrombosis is very dangerous, and anticlotting treatment must be started as soon as possible. Information on how thromboembolism occurs was described as early as the 19th century by the German physician Rudolf Virchow, who identified three main factors for the development of thrombosis (Figure 1).

Myeloproliferative diseases specifically are *PV*, essential thrombocythemia (*ET*) and primary myelofibrosis. All these diseases are associated with an overproduction of blood elements.

A large number of patients with myeloproliferative disorders have been found to carry a dominant *JAK2* mutation. This gene causes the expansion of hematopoietic progenitors in these disorders [1]. Furthermore, *JAK V617F* has been found to be a somatic mutation of hematopoietic cells. The *JAK V617F* mutation is most commonly found in patients with *PV*. Tefferi et al. [2] discussed the World Health Organization (WHO) criteria for the diagnosis of *PV*, *ET*, and primary myelofibrosis. All these diseases were described in their research using the older criteria and then described using the new proposed criteria. Baxter et al. [3] discussed acquired *JAK2* tyrosine kinase mutations in human myeloproliferative diseases. They noted that finding the *Val617Phe* (point mutation of Janus kinase 2) mutation may become a good identifier to distinguish myeloproliferative diseases from other similar ones. Levine et al. [4] reported how *JAK2* tyrosine kinase mutations are activated in myeloproliferative diseases. As a result, we found that inhibition of *JAK2V617F* leads to a reduction in hematopoietic cell proliferation. This tyrosine kinase is a potential target for drug therapy. Neunteufl et al. [5] described endothelial dysfunction in patients with *PV*. This endothelial dysfunction should be considered as a pathology in arterial thrombosis in polycythemia vera. Furthermore, Bonetti et al. [6] described the production of nitric oxide and the protection of the blood vessels against platelet and leukocyte adhesion. Th study demonstrated that polycythemia vera is associated with endothelial dysfunction and may cause other arterial diseases. The review also discussed endothelial dysfunction as a feature of atherosclerotic risk factor [7]. Its dysfunction leads to impaired endothelium-dependent vasodilation. Endothelial nitric oxide synthase in coronary artery disease was also described. Polymorphisms in *exon 7* of the *eNOS* (Endothelial NOS) gene are being studied. Finazzi et al. [8] discussed the risk of thrombosis in patients with *ET* and *PV* according to the *JAK2V617F* mutation. Due to the *JAK2V617F* mutation, *ET* is divided into two types. Ihle and Gilliland [9] compared the risk of thrombosis in *ET* and *PV* according to *JAK2* mutation status. The regulation of *JAK2* kinase activity was also summarized, followed by the activation of *JAK2* kinase by chromosomal translocation and mutations in myeloproliferative diseases. *JAK2V617F* is not the only genetic contributor to the disease phenotype. Again, the work proposed by Lippert et al. [10] dissected the observed group into the three diseases already mentioned and studied homozygosity and heterozygosity for *JAK2* mutations. A brief work reported by Pietra et al. [11], also examined somatic *JAK2 exon 12* mutations in patients with *JAK2* negative myeloproliferative disease. All patients with *exon 12* mutations had low erythropoietin levels. In total, eight different types of mutations and two new duplications were found in this study. Napoli and Ignarro [12] described nitric oxide and atherosclerosis, specifically their relationship. The physiological state is maintained in the bloodstream by laminar or turbulent flow. *NO* is also an inhibitor of blood elements that could bind to the vessel wall. Schwentker et al. [13] tried to answer the question of what role cytokines and nitric oxide play in tissue repair and injury. When *NO* enzymes are genetically deleted or pharmacologically attenuated, the organism’s wound healing is impaired. They described the roles of *NO* and cytokines in healing. Vallet [14] discussed vascular reactivity and tissue oxygenation. Injury to the blood circulation may imply a disruption of *O_2_* body regulation, and consequently, pathological restriction may occur.

Myeloproliferative neoplasms are clonal hematopoietic stem cell malignancies characterized by independence or hypersensitivity of hematopoietic progenitors to numerous cytokines. The molecular basis of most myeloproliferative neoplasms is unknown. *PV* is an acquired myeloproliferative neoplasm, characterized by the presence of polyglobulia variously associated with thrombocytosis, leukocytosis, and splenomegaly. *PV* progenitors are hypersensitive to erythropoietin and other cytokines [15,16,17]. *PV* has an incidence rate of 1.0 case per 100,000 people (prevalence rate of 44–57 per 100,000 people) and is a disease of the elderly, with a median age at diagnosis of 61 years. The median survival of *PV* is 18.9 years. *PV* has a chronic course, with thrombosis representing a major cause of morbidity and mortality. Less common causes include transformation to myelofibrosis and acute leukemia [18]. When *PV* is present, determining the peripheral blood hematocrit, or hemoglobin, will not accurately reflect the actual volume of red blood cells in the body because in *PV*, in contrast to other disorders causing erythrocytosis, the increase in red cell mass is usually associated with an increase in plasma volume [19]. Splenomegaly and myelofibrosis often occur in *PV* patients. Almost all *PV* patients harbor a mutation in the *JAK2* gene, mainly a *JAK2 V617F* point mutation [20]. Bone marrow morphology remains the cornerstone of diagnosis. The presence of a *JAK2* mutation is expected in *PV*, while approximately 90% of patients with *ET* express mutually exclusive *JAK2*, *CALR* (calreticulin), or myeloproliferative leukemia mutations [21,22]. *PV*, *ET*, and idiopathic myelofibrosis are clonal myeloproliferative neoplasms arising from a multipotent progenitor. The loss of heterozygosity (*LOH*) on the short arm of chromosome 9 (*9pLOH*) in myeloproliferative neoplasms suggests that 9p harbors a mutation that contributes to the clonal expansion of hematopoietic cells in these diseases [1]. The molecular pathogenesis of these entities is unknown, but tyrosine kinases have been implicated in several related disorders. The role of the cytoplasmic tyrosine kinase *JAK2* in patients with a myeloproliferative disorder has been extensively investigated [3]. *PV* increases the risk of arterial and venous thromboembolic complications. It was demonstrated that *PV* is associated with endothelial dysfunction in the preclinical phase of arterial disease [5]. Endothelial dysfunction is a systemic disorder that plays an important role in the pathogenesis of atherosclerosis. Current evidence suggests that endothelial status is determined not only by the individual risk factor burden. Rather, it may be regarded as an integrated index of all atherogenic and atheroprotective factors present in each individual [6]. The laboratory and clinical findings of 179 patients with *ET* and 77 with *PV* were classified according to the presence of the *JAK2 V617F* mutation and compared. The relationship between patients with *JAK2* wild-type *ET*, *JAK2 V617F* positive *ET* and *PV* (all with the *JAK2* mutation) was determined. The rate of thrombotic complications in *JAK2*-positive ET was significantly higher than in wild-type *ET* and not statistically different from that of *PV* patients [8]. The allelic frequency of the *JAK2-V617F* mutation in DNA and the expression levels of the mutant and wild-type *JAK2 mRNA* were determined in the granulocytes from 60 patients with *ET* and 62 patients with *PV* at the time of diagnosis. Using an allele-specific quantitative polymerase chain reaction (*qPCR*), *JAK2-V617F* was detected in 75% of *ET* and 97% of *PV* patients at diagnosis [10].

This present paper analyzes anonymized data from a clinical information system. Individuals were screened for high erythrocyte counts in the bloodstream and typical manifestations of *PV* disease. However, this disease was not detected in them (most of the subjects had the *JAKV617F* parameter without mutation), so the patients could not be diagnosed with primary myeloproliferative disease.

**Figure 2:**
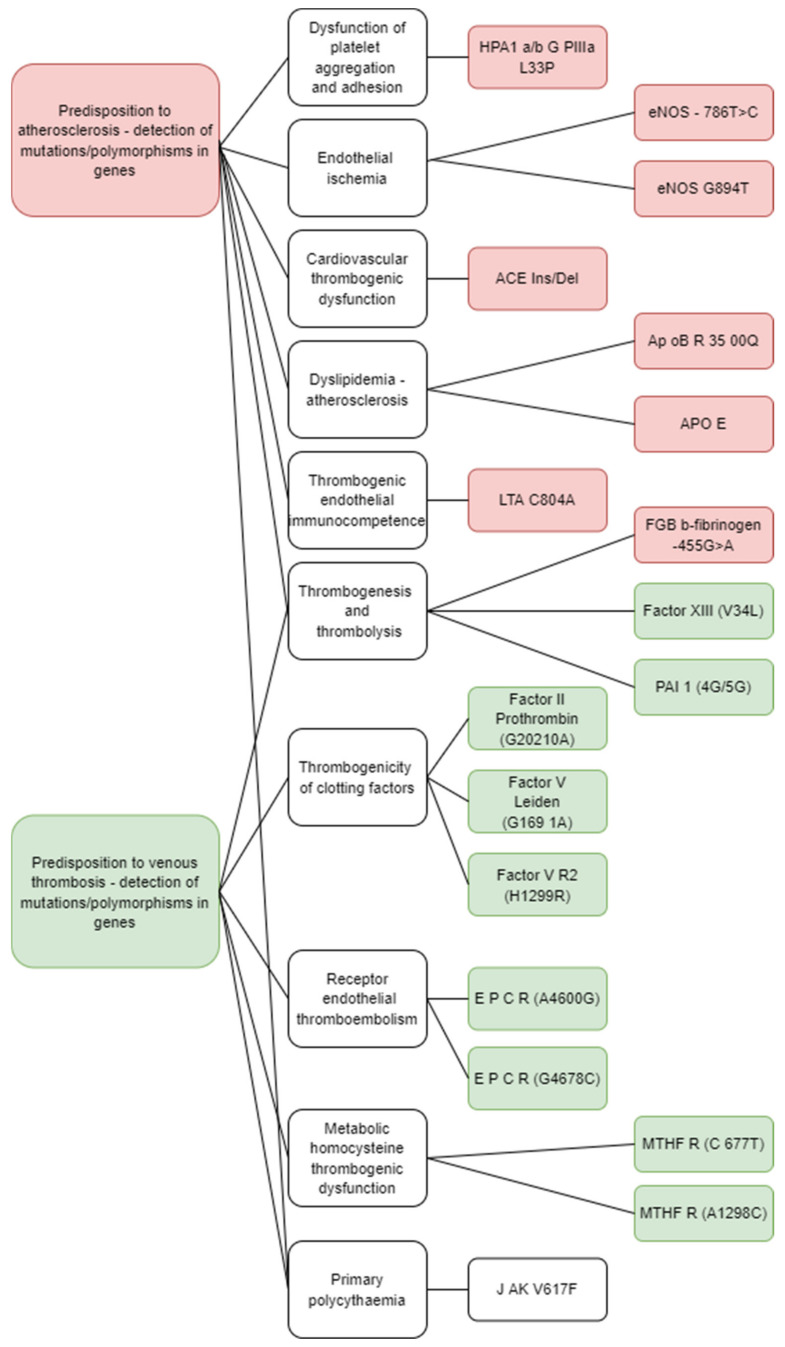
Investigated parameters within the whole research.

As summarized in Figure 2, in this work we tested coagulation factors (Factor *V Leiden*, Factor *V R2*, Factor II) as well as proteins responsible for the risk of atherosclerosis (*ApoB R3500Q, APO E*); endothelial ischemia (*eNOS-786T>C*, *eNOS G894T*); cardiovascular thrombogenic dysfunction (*ACE Ins/Del*); metabolic homocysteine thrombogenic dysfunction (*MTHFR A1298C*, *MTHFR C677T*); endothelial receptor thromboembolism (*EPCR A4600G*, *EPCRG4678C*); thrombogenic endothelial immunocompetence (*LTA C804A*); dysfunctional platelet aggregation and adhesion (*HPA1 a/b GPIIIa L33P*) and protein response during thrombogenesis and thrombolysis (*FGB b-fibrinogen −455G>A*, *Factor XIII, PAI-1 4G/5G*).

## 2. Materials and Methods

**Figure 3:**
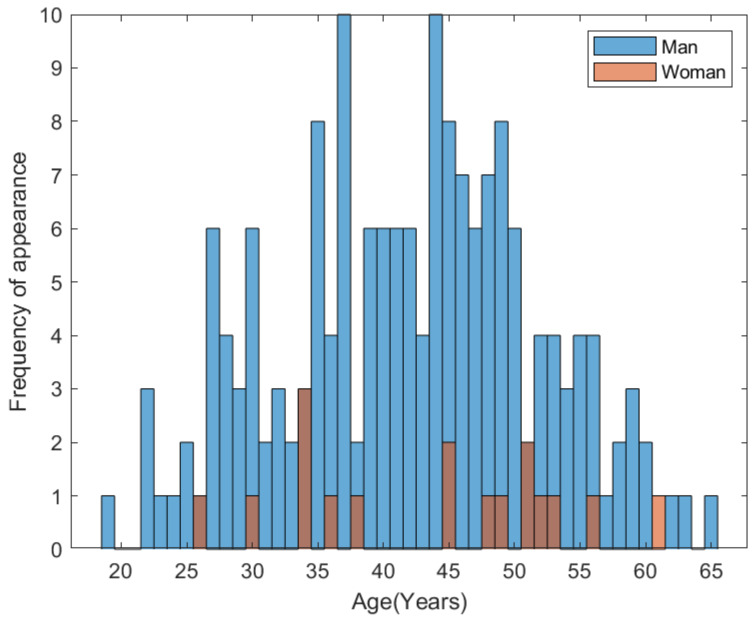
Dependence of age, frequency and sex.

The starting point of this study was based on a large cohort (approximately. 3000 subjects) of active blood donors, who did not demonstrate any clinical symptoms that would exclude them from actively donating blood. This study was approved by the ethical committee of University of Hradec Kralove on 16 August 2021 with the evidence number: 11/2021 (The complete report of the ethical committee can be found in the section: Institutional Review Board Statement). The donors also did not report any medical problems in their questionnaire. Based on transfusion medicine legislation, donors, among other parameters, mandatorily undergo hemoglobin level determination. A total of 190 subjects with repeated hemoglobin levels corresponding to polyglobulia were selected from the initial cohort (182 men and 17 women) and included in the study sample (Figure 3). The men ranged in age from 19 to 65 years and the women from 26 to 61 years. All subjects underwent genetic testing for defined parameters (thrombophilic mutations using real-time *PCR*, the *V614F* mutation of the *JAK-2 gene–PV*, and determination of predispositions to atherosclerosis using *PCR* and reverse hybridization). This studied cohort of selected donors was typical in its homogeneity, whereby phenotypic signs of the homozygous, heterozygous, or so-called wild-type form, i.e., without mutation, were found in all those included. For the selected subjects, the laboratory hemoglobin levels appeared to be possible signs of myeloproliferative.

To arrange the blood results in a comprehensible and clear manner, an overall data library was created in Excel. This was later imported into MATLAB R2019a, where data analysis with evaluation of individual parameter frequency was performed. Typified groups were thus created, and parameters with similar properties were unified. Data mining was used to process the data, whereby a contingency table of all possible mutation combinations was acquired. Parameters with a value close to zero were excluded as clinically nonsignificant. All parameters were then divided according to their correlations and their frequency, i.e., incidence, into three groups (without mutation, mutation at a ratio of 1:1 and predominantly heterozygous detection). Two hypotheses were defined as follows: the combination of homozygous and heterozygous predispositions carry a clinically more significant risk than a homozygous predisposition; homozygous predispositions carry a clinically higher risk than heterozygous predispositions.

### 2.1. Instrumentation for Analysis

Genetic predispositions to atherosclerosis were determined with the help of reverse hybridization, using CVD Strip Assay commercial kit (ViennaLab Diagnostics GmbH, Austria) and Applied Biosystems™ ProFlex™ PCR System (Thermo Fisher Scientific Inc., Waltham, MA, USA). Genetic testing of predisposition to venous thrombosis was based on the method of allelic discrimination TaqMan^TM^ SNP genotyping Assays (Thermo Fisher Scientific Inc., Waltham, MA, USA) and Applied Biosystems^TM^ 7300 Real-Time PCR System (Thermo Fisher Scientific Inc., Waltham, MA, USA). The *V617F* mutation in the *JAK2* gene was determined using Realquality *RS-JAK-2 V617F* (AB Analitica^®^, Padua, Italy) and Applied Biosystems^TM^ 7300 Real-Time PCR System.

### 2.2. Investigated Parameters

#### 2.2.1. Risk of Developing Atherosclerosis—*ApoB*: Familial Defective Apolipoprotein B-100; *ApoE*

Apolipoprotein B-100 is one of the forms of apolipoprotein B (*ApoB*). It represents the basic protein component of low (*LDL*) and very low (*VLDL*) lipoproteins. It plays an important role in the transport of lipids and cholesterol. The *R3500Q* mutation is one of the most widespread mutations involving the *ApoB* gene [23]. It is caused by the substitution of the amino acid base *G* (guanine) for *A* (adenine) in *exon 26*. This leads to the substitution of arginine with glutamine at position 3527, leading to a change in the protein structure at the site of receptor binding. This results in a lower affinity of *LDL* particles to receptors and their subsequent accumulation in the blood. The familial genetic defect of *ApoB* is one of the causes of familial hypercholesterolemia. Accumulation of cholesterol in the blood significantly increases the risk of atherosclerosis and myocardial infarction. The frequency of heterozygotes is 1:500, and the frequency of mutated homozygotes is 1:1,000,000 [24,25]. Apolipoprotein *E* (*ApoE*) is a component of *VLDL*, and its main task is to remove excess cholesterol from the blood and transport it into the liver for further processing [26]. It ensures that cholesterol levels remain physiological and thus significantly participates in the prevention of cardiovascular disease. Three commonly occurring alleles of the *ApoE* gene have been identified—*ApoE2*, *ApoE3* and *ApoE4*. The *E2/E2* homozygote is exposed to a higher risk of familial hyperlipoproteinemia (type III). In contrast, the *ApoE4* isoform is a factor for atherosclerosis because of the increased level of total cholesterol, both in the heterozygous and especially in the homozygous form. The *E4* allele has been identified as a significant and independent (on age, gender, and atherosclerotic disease) predictive factor for the development of aortic stenosis [27,28].

#### 2.2.2. Coagulation Factors and Their Mutations—Factor *V Leiden*, Factor *V R2*, Factor II

Mutations of coagulation factors are often associated with a higher risk of thrombophilia. Under physiological conditions, the coagulation cascade ensures the formation of blood clots. Factor *V Leiden* (*G1691A*) is the most common genetic predisposition for thrombosis. This point mutation induces resistance to the anticoagulant activity of *APC* (activated protein *C*) in factor *V*, and this consequently prolongs the process of thrombosis. The Leiden mutation is the most frequent congenital predisposition for abnormal blood clotting. Heterozygotes are more frequent in the population than homozygotes. The risk of developing thromboembolism is 3–8× higher in heterozygotes than in the unmutated population, and it is 50–80× higher in homozygotes [29,30,31]. Factor *V R2* (*H1299R*) is another genetic mutation of factor V. It is induced by polymorphism *A4070G* in *exon 13*, leading to the substitution of histidine (*R1 allele*) for arginine (*R2 allele*) at *1299 B* of the domain. This substitution leads to a decrease in factor *V* levels and its resistance to activated protein *C* (*APC*) [32]. Factor II prothrombin (*G20210A*) is one of the most frequent genetic thrombophilic predispositions in the European population and plays a significant role in venous thrombosis. Together with factor *V*, prothrombin is part of the coagulation cascade ensuring correct blood clotting [33]

#### 2.2.3. Endothelial Ischemia—*eNOS* (*−786T>C*), *eNOS (G894T)*

Endothelial nitric oxide synthase (*eNOS*) is one of the three isoforms of nitric oxide (*NO*) synthesis that demonstrate sequence and functional homology. The gene for *eNOS* (that is, *NOS3*) is located on chromosome *7q35-36*. *NO* formation is catalyzed by endothelial *NO* synthase. It has vasodilating, anti-inflammatory, and antiproliferative properties. Decreased production of *NO* may, among others, lead to smooth muscle cell proliferation. Moreover, *eNOS* (*G894T*) polymorphism for the *NO* synthase gene has been associated with the incidence of coronary artery spasms and myocardial infarction and in some cases has been a predictor of restenosis. In contrast, *eNOS* (*G894T*) polymorphism in the same gene is a predictor of serious coronary events without increasing the risk of in-stent restenosis. Similarly, another polymorphism in the promoter region of the *NOS3* gene (*−786T>C*) affects the expression of this gene, increasing susceptibility to coronary disease [34,35,36].

#### 2.2.4. Cardiovascular Thrombogenic Dysfunction (*ACE* Ins/Del)

Angiotensin converting enzyme (*ACE*) plays an important role in the regulation of blood pressure as part of the renin–angiotensin system. *ACE* is present on the endothelial cells in many tissues (e.g., the uterus, placenta, heart, brain, kidneys, leukocytes, alveolar macrophages, peripheral monocytes and neurons). It enables the conversion of angiotensin I to the vasoconstrictive-aldosterone-stimulating peptide angiotensin II (*ATII*) which participates in vasoconstriction [37,38].

#### 2.2.5. Metabolic Homocysteine Thrombogenic Dysfunction–*MTHFR A1298C*, *MTHFR C677T*

The 5,10-metlylenetetrahydrofolate reductase (*MTHFR*) enzyme is a key enzyme for the metabolism of homocysteine. Point mutations in the *MTHFR* gene lead to the formation of an enzyme with increased thermolability and decreased activity, and this significantly correlates with an increased level of homocysteine in plasma. Homocysteine may thus contribute to the development of atherosclerosis and thrombosis because of changes in vascular cell proliferation and promotion of prothrombotic activities within the vascular wall. The most frequent and best-known mutations of the *MTHFR* gene involve substitution of *C* for *T* at position 677 (*C677T*). The second most frequent mutation involves the substitution of *A* for *C*, at position 1298 (*A1298C*). Approximately 30% of homozygotes and 65% of heterozygotes with the *C677T* mutation demonstrate *MTHFR* activity. The *A1298C* mutation in both the homozygous and heterozygous form, per se, does not significantly alter plasma levels. However, a compound heterozygote with *C677T* becomes a risk factor for milder hyperhomocysteinemia [39,40].

#### 2.2.6. Endothelial Receptor Thromboembolism—*EPCR A4600G*, *EPCR G4678C*

*EPCR* is an endothelial protein C receptor predominantly located on the endothelium of large vessels. Its physiological role consists of localizing protein *C* in order to be activated by the thrombin–thrombomodulin complex. It occurs as the *A3* haplotype (*A4600G*) and the *A1* haplotype (*G4678C*). Individuals with the *A3* haplotype have higher plasma levels of *EPCR* and are thus at risk of venous thrombosis. In contrast, a protective effect against venous thromboembolism may manifest in individuals with the *A1* haplotype [41,42].

#### 2.2.7. Thrombogenic Endothelial Immunocompetence—*LTA C804A*

Lymphotoxin-alfa (*LTA*) is a representative of the tumor necrosis family of cytokines and was originally isolated on the basis of its antitumor activity. Later on, its anti-inflammatory and immunological activities were demonstrated. The *LTA* gene is located on chromosome *6p21.3*. *LTA* as an inflammatory cytokine is expressed in atherosclerotic lesions, and it plays a leading role in the development of atherosclerosis (the greatest risk factor for arterial accidents). In this context, an association has been found between the *C804A* (*T26N*) polymorphism for the *LTA* gene and coronary and cerebrovascular accidents [43].

#### 2.2.8. Dysfunctional Platelet Adhesion and Aggregation—*HPA1 a/b GPIII L33P*

The *HPA 1 (a/b)–GPIIIa (L33P)*–platelet glycoprotein complex IIb/IIIa (*a2bb3*) plays a role in cell interactions and includes binding sites for fibrinogen, the von Willebrand factor, fibronectin and vitronectin. Polymorphisms in the genes coding these glycoprotein complexes may affect many processes in the human body (resistance to aspirin, cardiovascular disease, changes in molecule antigenic properties possibly leading to posttransfusion purpura, life-threatening thrombocytopenia and neonatal thrombocytopenic purpura). The most common and clinically significant alleles of the *GPIIIa* gene are *P1A1* (*HPA-1a*) and *P1A2* (*HPA-1b*). *HPA-1b* may in some cases increase platelet aggregation. Heterozygotes and homozygotes for *HPA-1b* more frequently develop thin-walled, vulnerable atherosclerotic plaques, prone to ruptures and subsequent massive thrombosis. However, carriers of the *HPA-1b* allele only have a mild risk of developing coronary artery disease, myocardial infarction, or restenosis following percutaneous interventions. Nonetheless, this risk increases significantly with the concomitant incidence of other polymorphisms such as, e.g., those for the *eNOS* and *PAI-1* genes. However, the presence of at least one allele of *HPA 1b* is currently considered to be one of the thrombophilic factors for pre-eclampsia in pregnant women [44,45].

#### 2.2.9. Thrombogenesis and Thrombolysis—*FGB b*-fibrinogen−*455G>A*, Factor XIII, *PAI-1 4G/5G*

*FGB b*-fibrinogen (*−55G>A*) is the coagulation factor I with a molecular weight of 340 kDa. This dissolvable glycoprotein is commonly found in blood plasma as well as in platelet granules. It is one of the key proteins of thermocoagulation. It plays a role in platelet aggregation and affects plasma viscosity. The presence of the A-allele (with an incidence of approximately 20% in the population) is associated with a significantly increased promoter activity of the gene and thus with increased fibrinogen plasma levels. The *−455G>A* polymorphism is significant in relation to fibrinogen levels in persons with coronary artery disease as well as in patients after revascularization bypass surgery [46]. Factor XIII (*V34L*), or fibrin-stabilizing factor, is a transglutaminase consisting of a tetramer of two *A* and two *B* units. The nucleotide substitution of amino acid bases *C* (cytosine) for *T* (thymine) in *exon 2* of the *F13A1* gene to the substitution of valine for leucine at position 34 of the peptide chain. Homozygotes for this mutation demonstrate a significantly higher activity of this enzyme than individuals without any mutation, while heterozygotes demonstrate an intermediate activity of this enzyme. It has been demonstrated that the Lue 34 variant has an important protective effect against venous thromboembolism [47]. *PAI-1 4G/5G* is a key inhibitor of tissue plasminogen activator (*tPA*) and urokinase (*uPA*) as well as of plasminogen activators and consequently of fibrinolysis. The *PLANH1* promotor gene coding for *PAI*- may include a polymorphism known as *4G/5G* whereby the *5G* allele is less transcriptionally active than the 4 G allele. Primary elevation of *PAI-1* levels is found in individuals with *4G/4G* polymorphism. It is assumed that this polymorphism is associated with a higher incidence of arterial thrombosis [48].

## 3. Results

Results of clinical evaluation for the studied subjects are summarized in the following Table 1 and Table 2.

Men of all ages predominated in the studied sample. In most individuals, the primary myeloproliferative neoplasm was not confirmed. This means that the subjects in this sample suffered from secondary *PV*.

The parameters were then divided according to the frequency of mutation incidence expressed in a percentage (heterozygote and homozygote). Thanks to this distribution, we were able to organize parameters into groups in which they occurred most frequently.

It was thus inferred that this was not the case of a primary myeloproliferative disease (given that the sample consisted of secondary nonautonomous *PV*). The last parameter was that of dyslipidemia–atherosclerosis involving the *Apo B R3500Q* genotype. The parameter involving the *Apo E* genotype occurred in the 20 to 50% group. It was thus inferred that the parameter was not clinically relevant for our needs. The 20 to 50% group included the parameter of platelet aggregation and adhesion dysfunction, i.e., *HPA1 a/b GPIIIa L33P*. This parameter was projected to occur in the group of greater than 50%.

**Figure 4:**
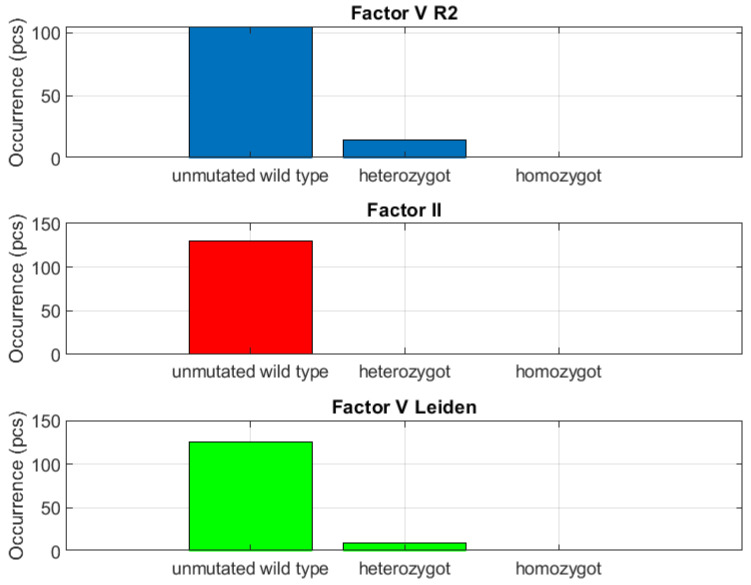
Clearly negative mutation capture. Group of clotting factor Thrombogenicity of is evaluated.

**Figure 5:**
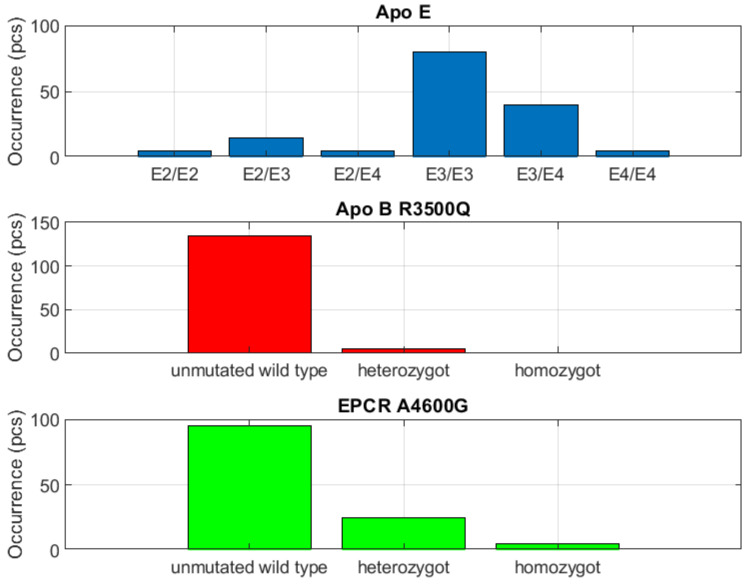
Clearly negative mutation capture. Here is dyslipidemia-atherosclerosis and receptor of endothelial thromboembolism *EPCR A4600G*.

The group with less than 20% incidence included coagulation factor thrombogenicity. This parameter occurred solely in the group with less than 20% incidence, and thus appeared to be clinically nonrelevant (Figure 4 and Figure 5). Coagulation factor thrombogenicity integrates factor *V Leiden*, factor *V R2* and factor *II*. This group also includes primary *JAK V617F* positive *PV*, which was demonstrated in only two subjects within the entire sample.

**Figure 6:**
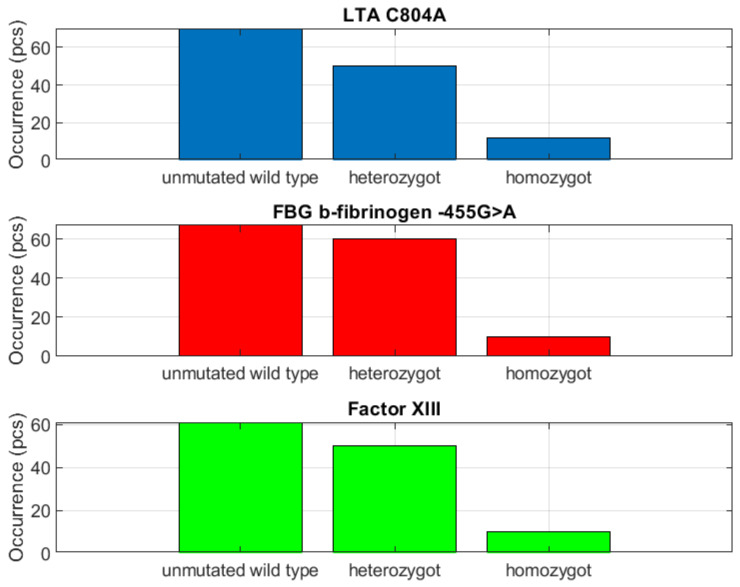
Heterozygous distribution and nonmutant alleles in a 1:1 ratio.

The question thus arises as to whether it is necessary to influence platelet function using antiplatelet agents or vice versa. The 20 to 50% group also included the thrombogenic endothelial immunocompetence *LTA C804A* parameter. It occurred in subjects at a ratio of 1:1 for heterozygosity and nonmutated allele, i.e., wild type, similar to factor *XIII* thrombogenesis and thrombolysis (Figure 6).

The parameter of endothelial receptor thromboembolism, i.e., *EPCR A4600G*, demonstrated unequivocally negative detection of the mutation. A 50% risk of mutation incidence was inferred based on the results of the 20 to 50% group. The question remains whether testing for these parameters clinical significance has, given that the 1:1 ratio will be less contributing than in the greater than 50% group. Individuals with a 50% incidence of mutations harbored endothelial ischemia and thrombogenesis and thrombolysis, i.e., *FGB b-fibrinogen*, at a ratio of 1:1 for heterozygosity and nonmutated allele.

**Figure 7:**
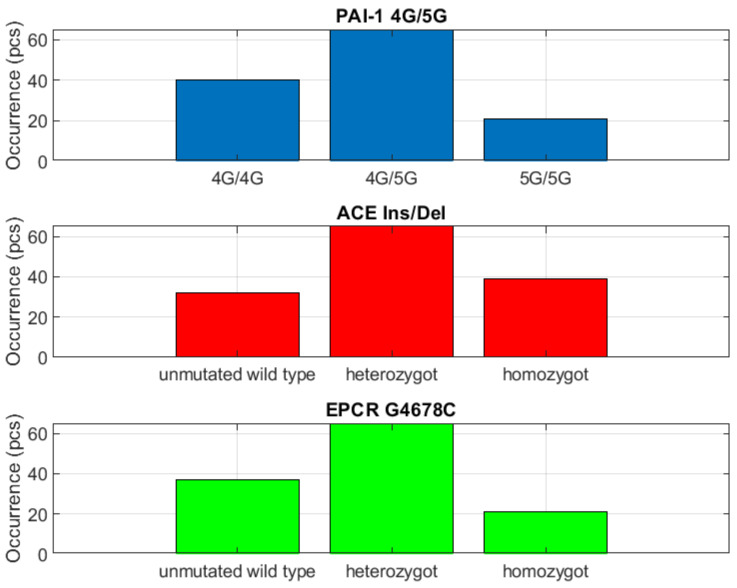
Dominating heterozygosity.

Other representative parameters of the thrombogenesis and thrombolysis, i.e., *PAI-1 4G/5G* and endothelial receptor thromboembolism, i.e., *EPCR G4678C*, were dominant on heterozygote detection (Figure 7).

One important finding was that the greater than 50% group included cardiovascular thrombogenic dysfunction *ACE ins/del*, which had not been given much attention previously.

**Figure 8:**
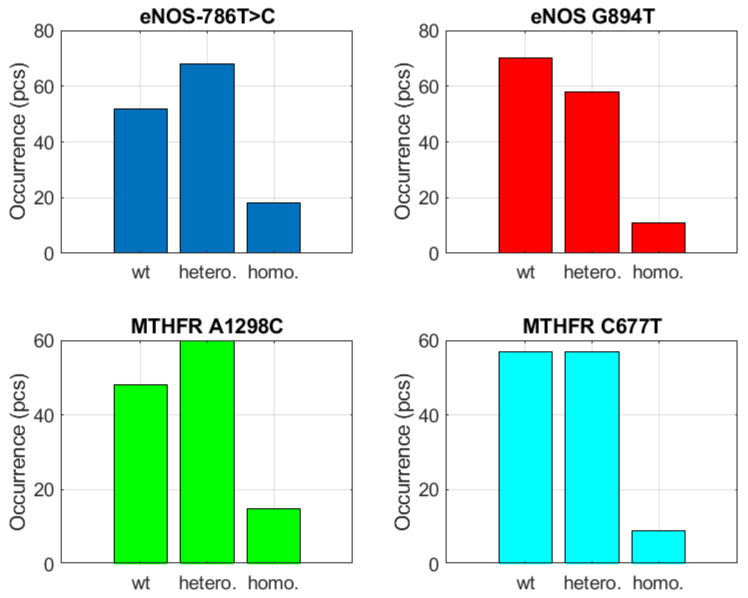
Heterozygous distribution and nonmutant alleles in a 1:1 ratio.

The last parameter was the metabolic homocysteine thrombogenic dysfunction, i.e., *MTHFR*, which occurred at a ratio of 1:1 for heterozygosity and nonmutated allele (Figure 8).

Interestingly, both studied *MTHFR* parameters were found only in the group with a greater than 50% incidence. In this group, the 1:1 distribution was clinically more significant than in the 20 to 50% group. It is possible to derive from these tables that the greater than 50% group, which includes cardiovascular thrombogenic dysfunction and metabolic homocysteine thrombogenic dysfunction are the most significant from a clinical aspect. This is followed by the 20 to 50% group. In the less than 20% group, endothelial thromboembolism, thrombogenesis and thrombolysis appear to be most significant.

### 3.1. Evaluation of the Group with Detection Less than 20%

The group with an incidence of less than 20% includes parameters such as primary *JAK V617F* positive *PV*, dyslipidemia–atherosclerosis and coagulation factor thrombogenicity. All these parameters appeared indeterminate in the analysis of the data. Based on results, it was not possible to determine whether it was a case of direct or indirect correlation. For this reason and because of their low incidence, these parameters were evaluated only marginally, and they appear nonsignificant for the clinical interpretation of this study.

### 3.2. Evaluation of the Group with Detection from 20 to 50%

The *APO E* parameter (dyslipidemia-atherosclerosis) in the *E3/E3* allele demonstrated indirect dependency for *eNOS G894T=homozygote* (endothelial ischemia) as well as for *EPCR A4600G=homozygote* (endothelial receptor thromboembolism), factor *XIII=homozygote* (thrombogenesis and thrombolysis), *HPA1 a/b GPIIIa L33P=a/b* (dysfunction of platelet aggregation and adhesion) and *LTA C804A=homozygote* (thrombogenic endothelial immunocompetence). The *APO E=E2/E2* parameter demonstrated a direct correlation with factor *XIII=homozygote*, which has a protective effect. The *eNOS G894T* parameter in the homozygous allele was in direct correlation only with factor *XIII=homozygote*, which reduces risk as homozygous factor *XIII* has a protective effect. The *eNOS G894T* in the nonmutated allele was indirectly dependent on *Apo E=E2/E2*, *HPA1 a/b GPIIIa L33P=a/b* (heterozygote) and *LTA C804A=homozygote* (thrombogenic endothelial immunocompetence). The *EPCR A4600G=homozygote* parameter was in direct correlation with *eNOS*.

The presence of *G894T=homozygote* and *LTA C804A=homozygote* indicate an acceleration risk. The *EPCR A4600G* parameter in the nonmutated wild-type allele was indirectly dependent on *APO E=E2/E2*, homozygote *eNOS G894T*, Factor *XIII*, *LTA C804A* and *HPA1 a/b GPIIIa L33P=a/b* (heterozygote).

Factor *XIII* was in direct correlation with *eNOS G894T=homozygote* and *LTA C804A=homozygote* (risk reduction). For the nonmutated and heterozygote allele, the parameter was indirectly correlated with homozygous *EPCR A4600G*. The HPA1 a/b GPIIIa L33P=a/a parameter was indirectly dependent on *Apo E=E2/E2* and the homozygous allele of *eNOS G894T, EPCR A4600G*, Factor *XIII* and *LTA C804*. Detected heterozygotes were in direct correlation with homozygous factor *XIII*, again reducing risk.

The last studied parameter in this group was *LTA C804A*. Homozygosity was in direct correlation with homozygous factor *XIII*. The nonmutated allele was indirectly dependent on *Apo E=E2/E2*, homozygous *eNOS G894T* and heterozygous *HPA1 a/b GPIIIa L33P*.

### 3.3. Evaluation of the Group with Detection Greater than >50%

The *ACE ins/del=ins/del* parameter (cardiovascular thrombogenic dysfunction) was significant when detected in the heterozygous form, where it was shown be indirectly dependent on *eNOS* *−786T>C=homozygote* (endothelial ischemia) and *FGB b-fibrinogen 455G>A=homozygote* (thrombogenesis and thrombolysis). If the *ACE* parameter is in the heterozygous form, the risk of endothelial ischemia and thrombogenesis and thrombolysis decreases. It was also shown that this *ACE ins/del=ins/ins* parameter was directly dependent on *EPCR G4678C=homozygote* (endothelial thromboembolism receptor), *MTHFR A1298C=homozygote*, *MTHFR C677T=homozygote* and *PAI-1 4G/5G=5G/5G*. In this case, we refer to an acceleration risk. The *eNOS* *−786T>C=heterozygote* parameter (endothelial ischemia) was significant in the case of heterozygosity, which was shown to be directly dependent on *ACE ins/del=ins/ins* (cardiovascular thrombogenic dysfunction) and *FGB b-fibrinogen −455G>A=homozygote* (thrombogenesis and thrombolysis). The homozygous form of *eNOS* *−786T>C* was also shown to be directly dependent on *EPCR G4678C*, *MTHFR A1298C*, *MTHFR C677T* and *PAI-1 4G/5G*, all in the homozygous form. The *eNOS* parameter in the homozygous form increases probability in the group of metabolic homocysteine thrombogenic dysfunction. The *EPCR G4678C* parameter (receptor endothelial thromboembolism) in the heterozygous form was indirectly correlated to *MTHFR A1298C=homozygote* and *MTHFR C677T=homozygote* (metabolic homocysteine thrombogenic dysfunction) as well as *PAI-1 4G/5G=5G/5G* (thrombogenesis and thrombolysis). The homozygous form of *EPCR G4678C* was significant because of its direct correlation with *ACE ins/del=ins/ins*, as well as homozygous *eNOS −786T>C* and *FGB b-fibrinogen* *−455G>A*. The *FGB b-fibrinogen* *−455G>A* parameter in its homozygous form was in direct correlation with homozygous *EPCR G4678C*, *MTHFR A1298C* and *C677T* as well as *PAI-1 4G/5G=5G/5G*. The wild-type allele of fibrinogen was then indirectly correlated to *ACE ins/del=ins/ins* and homozygous *eNOS* *−786T > C*. The *MTHFR A1298C* homozygous parameter was directly correlated with *ACE ins/del=ins/ins*, *eNOS −786T>C=homozygote* and *FGB b-fibrinogen* *−455G> A=homozygote*. Heterozygous *MTHFR A1298C* was indirectly correlated with homozygous *EPCR G4778C*, *MTHFR C677T* and *PAI-1 4G/5G*. The *MTHFR C677T=homozygote* parameter was directly correlated with *ACE ins/del=ins/ins* (this result was highly significant for cardiogenic risk), *eNOS* *−786T>C=homozygote* and *FGB b-fibrinogen455G>A=homozygote*. *MTHFR C677T* heterozygote demonstrated an indirect correlation with homozygous *EPCR G4678T*, *MTHFR A1298C* and *PAI-1 4G/5G=5G/5G*. The *PAI-1 4G/5G=4G/5G* was indirectly correlated with the homozygous allele of *EPCR G4678C*, *MTHFR A1298C*, *MTHFR C677T* and *PAI-1 4G/5G=5G/5G*. The homozygous *PAI-1* allele was directly correlated to *ACE ins/del=ins/ins*, homozygous *eNOS −786T>C* and *FGB b-fibrinogen *−455G>A**.

## 4. Discussion and Conclusions

This research aimed to analyze the results of genetic thrombogenic and atherogenic parameters in selected donors (predominantly men) who had higher levels of hemoglobin but no diagnosis of the chronic progressive myeloproliferative disease, *PV*. This disease predominantly involves mutations of the *JAK V617F* gene. Data were acquired from Nový Jičín Hospital, in the Czech Republic. The study sample was a homogenous group of 199 patients aged between 19 and 65 years who had high levels of hemoglobin and physical symptoms of *PV*, and in whom a primary myeloproliferative disorder had not been demonstrated.

The most contributive groups included endothelial ischemia, cardiovascular thrombogenic dysfunction, metabolic homocysteine thrombogenic dysfunction, thrombogenesis and thrombolysis and endothelial receptor thromboembolism. All the parameters in these groups were clinically relevant. The *ACE ins/del* parameter and cardiogenic risk demonstrated great significance. This enzyme, usually synthesized by endothelial cells, regulates blood pressure (smooth muscle constriction and subsequent arterial narrowing). If *ACE* was present in the heterozygous form, the thrombogenic risk in endothelial ischemia, as well as thrombogenesis and thrombolysis, decreased. In the homozygous form, the risk increased in relation to the parameters *MTHFR, EPCR G4678C* and *PAI-1* (all in the homozygous form). Clinically relevant parameters were detected in the endothelial ischemia group. The parameters studied included *eNOS G894T* and *eNOS* *−786T>C*. Both these oxides have vasodilating, anti-inflammatory and antiproliferative properties. Mutations involving these parameters lead to smooth muscle proliferation, which may lead to coronary artery disease. The *eNOS* *−786T>C* parameter in the homozygous form increased the risk in the case of homocysteine. Furthermore, homozygosity for *eNOS G894T*, *EPCR A4600G* and *LTAC804A* demonstrated an acceleration risk. Metabolic homocysteine thrombogenic dysfunction was the only one to occur in the greater than 50% group. Mutations of these genes lead to changes in DNA that subsequently increase blood homocysteine levels, leading to cardiovascular disease or thrombosis. *MTHFR A1298C* in combination with *MTHFR C677T* was a risk factor. The *MTHFR C677T* homozygote parameter with *ACE ins/ins* represented a major cardiogenic risk. Endothelial receptor thromboembolism *EPCR* occurred in the 20 to 50% group as well as in the greater than 50% group. This endothelial receptor most frequently occurs on the vascular endothelium. *EPCR A4600G* in its homozygous allele carried an acceleration risk with *eNOS G894T* and *LTA C804A*. Conversely, *EPCR G4678C* clinically decreased the inference of thrombogenic risk in the group of metabolic homocysteine thrombogenic dysfunction. The last parameter was *PAI-1 4G/5G*, a regulator of homeostasis that inhibits the plasminogen activator. The whole study included 52% of heterozygotes and 16% of homozygotes for this gene. Homozygosity increased the risk in relation to cardiovascular thrombogenic dysfunction, endothelial receptor thromboembolism and homocysteine thrombogenic dysfunction. *ACE ins/del* (cardiovascular thrombogenic dysfunction) appeared to be an unequivocal parameter, one that was mutated in all groups and thus carried an acceleration risk. Based on the results, groups with a higher incidence of mutations were assessed as presenting a greater risk for patients with regards to *PV*. Among the studied patients, it was not possible to determine unequivocally who had a higher risk of developing this disease, as the individual combinations of mutations could manifest as different symptoms of the disease. In general, however, it was possible to state that the whole studied sample was at risk in the presence of manifestations of *PV* and no unequivocal diagnosis. The group of less than 20% incidence appeared to be clinically nonsignificant for the *PV* investigation and therefore not testing irrelevant mutations could prove cost-effective. Conversely, the *JAK V617F* parameter from this group must be tested, as it can unequivocally rule out or confirm *PV* as the primary disease.

The above results obtained through the present study helped to select appropriate parameters for the prediction of myeloproliferative disease, which has not been previously genetically confirmed in patients who show signs of this disease. Research in this area will be continued and a statistical sample of patients with signs of the disease will be expanded on which to validate selected parameters that could be used to detect the disease. Further extensive meta-analysis will be performed on these data samples.

## Figures and Tables

**Table 1:** Percentage evaluation of results in the table.

<20% with Mutation	20–50% with Mutation	>50% with Mutation
*JAK* V617F	eNOS G894T	eNOS-*786T>C*
Apo B R3500Q	LTA C804A	ACE Ins/Del
Factor *V Leiden*	HPA1 a/b GPIIIa L33P	*FGB b*-fibrinogen −*455G>A*
Factor *V R2*	Factor XIII	MTHFR C677T
Factor II	EPCR A4600G	EPCR G4678C
-	Apo E	MTHFR A1298C
-	-	PAI-1 4G/5G

**Table 2:** Percentage evaluation of results in table after distribution.

<20% with Mutation	20–50% with Mutation	>50% with Mutation
Primary PV	Endothelial ischemia	Endothelial ischemia
Dyslipidemia-atherosclerosis	Thrombogenic endothelial immunocompetence	Cardiovascular thrombogenic dysfunction
Thrombogenicity of clotting factors	Dysfunction of platelet aggregation and adhesion	Thrombogenesis and thrombolysis
Thrombogenicity of clotting factors	Thrombogenesis and thrombolysis	Metabolic homocysteine thrombogenic dysfunction
Thrombogenicity of clotting factors	Receptor endothelial thromboembolism	Receptor endothelial thromboembolism
-	Dyslipidemia- atherosclerosis	Metabolic homocysteine thrombogenic dysfunction
-	-	Thrombogenesis and thrombolysis

## Data Availability

All the data, which are used in this study can be found in the link: https://www.dropbox.com/sh/s5vs8eh2wrwqogo/AAAXZs6IkJpjBTvC1XNwrw2Wa?dl=0.
